# Traumatized triad of complementopathy, endotheliopathy, and coagulopathy ˗ Impact on clinical outcomes in severe polytrauma patients

**DOI:** 10.3389/fimmu.2022.991048

**Published:** 2022-10-20

**Authors:** Zhangsheng Yang, Tuan D. Le, Milomir O. Simovic, Bin Liu, Tamara L. Fraker, Tomas S. Cancio, Andrew P. Cap, Charles E. Wade, Jurandir J. DalleLucca, Yansong Li

**Affiliations:** ^1^ United States Army Institute of Surgical Research, Joint Base San Antonio Fort Sam Houston, TX, United States; ^2^ Trauma Immunomodulation Program, The Geneva Foundation, Tacoma, WA, United States; ^3^ Department of Surgery, University of Texas Health McGovern Medical School, Houston, TX, United States; ^4^ Scientific Research Department, Armed Forces Radiobiological Research Institute, Bethesda, MD, United States

**Keywords:** trauma, complementopathy, endotheliopathy, coagulopathy, intercommunication, clinical outcomes, endotype, phenotype

## Abstract

Complementopathy, endotheliopathy, and coagulopathy following a traumatic injury are key pathophysiological mechanisms potentially associated with multiple-organ failure (MOF) and mortality. However, the heterogeneity in the responses of complementopathy, endotheliopathy, and coagulopathy to trauma, the nature and extent of their interplay, and their relationship to clinical outcomes remain unclear. Fifty-four poly-trauma patients were enrolled and divided into three subgroups based on their ISS. Biomarkers in blood plasma reflecting complement activation, endothelial damage, and coagulopathy were measured starting from admission to the emergency department and at 3, 6, 12, 24, and 120 hours after admission. Comparative analyses showed that severely injured patients (ISS>24) were associated with longer days on mechanical ventilation, in the intensive care unit and hospital stays, and a higher incidence of hyperglycemia, bacteremia, respiratory failure and pneumonia compared to mildly (ISS<16) or moderately (ISS=16-24) injured patients. In this trauma cohort, complement was activated early, primarily through the alternative complement pathway. As measured in blood plasma, severely injured patients had significantly higher levels of complement activation products (C3a, C5a, C5b-9, and Bb), endothelial damage markers (syndecan-1, sTM, sVEGFr1, and hcDNA), and fibrinolytic markers (D-dimer and LY30) compared to less severely injured patients. Severely injured patients also had significantly lower thrombin generation (ETP and peak) and lower levels of coagulation factors (I, V, VIII, IX, protein C) than less severely injured patients. Complement activation correlated with endothelial damage and hypocoagulopathy. Logistic regression analyses revealed that Bb >1.57 μg/ml, syndecan-1 >66.6 ng/ml or D-dimer >6 mg/L at admission were associated with a higher risk of MOF/mortality. After adjusting for ISS, each increase of the triadic score defined above (Bb>1.57 µg/ml/Syndecan-1>66.6 ng/ml/D-dimer>6.0mg/L) was associated with a 6-fold higher in the odds ratio of MOF/death [OR: 6.83 (1.04-44.96, P=0.046], and a 4-fold greater in the odds of infectious complications [OR: 4.12 (1.04-16.36), P=0.044]. These findings provide preliminary evidence of two human injury response endotypes (traumatized triad and non-traumatized triad) that align with clinical trajectory, suggesting a potential endotype defined by a high triadic score. Patients with this endotype may be considered for timely intervention to create a pro-survival/organ-protective phenotype and improve clinical outcomes.

## Introduction

Trauma is the leading cause of death, especially for individuals aged 1-44 (1). In the US, trauma accounts for 2.8 million hospitalizations, 214,000 deaths, and costs of $671 billion annually (1). The pathophysiological mechanisms of trauma are highly intricate, and early innate immune dysfunction plays a significant role (2–4). Acute severe trauma along with damage-associated molecular patterns (DAMPs) (5) and pathogen-associated molecular patterns (PAMPs) (4, 6, 7), and post-trauma early therapeutic approaches (blood product transfusion, ECLS devices, damage control surgery, ventilation, volume resuscitation, etc.) (8, 9) rapidly activates the intravascular innate immune system consisting of the plasma cascade systems (complement (10), coagulation (11), and the contact system (12)), blood cells (2, 4), and endothelial cells (2, 4), ultimately leading to trauma-induced thromboinflammation, multiple-organ failure (MOF), and mortality (2, 4, 6–8, 13). Although there has been a significant advancement in trauma care, trauma treatment remains an enormous challenge, especially in the early phase after injury, as the underlying pathophysiological mechanisms associated with such traumatic injuries are not yet fully understood.

The complement is one of the vital pathological mediators involved in trauma (10, 14, 15). After severe trauma complement is activated promptly, as evidenced by a rapid reduction in complement hemolytic activity due to complement consumption, and a significant elevation of complement activation products at both the systemic level and within local tissues. Ultimately, the combination of complement consumption and activation causes the development of complementopathy (10, 16). Trauma-induced complementopathy, as well as other innate immune disorder, further drives whole-body inflammation, which is clinically manifested as systemic inflammatory response syndrome (SIRS), compensatory anti-inflammatory response syndrome (CARS), and persistent inflammation, immunosuppression, and catabolism syndrome (PICS) (17–19) disorders, all of which provoke the development of MOF, in turn leading to death if its causes remain uncontrolled (20).

Severe trauma, especially when patients are in hemorrhagic shock, causes endothelial cell damage and a loss of the integrity of the endothelium, leading to endothelial dysfunction (endotheliopathy) (21, 22). The endothelial layer comprises endothelial cells, the basal lamina, and the endothelial glycocalyx coating the luminal surface (23). After severe trauma, the endothelial glycocalyx would often shed off, usually because of DAMPs, neutrophil extracellular traps, hypovolemia, and ischemia/reperfusion injury (IRI) (24). Syndecan-1 and soluble thrombomodulin (sTM) are two frequently used biomarkers reflecting endothelial damage (25). Particularly, syndecan-1, a heparin-sulfate proteoglycan, is currently considered a key marker of endothelial glycocalyx degradation (26). Thrombomodulin, an anticoagulant protein expressed on the endothelial surface, activates the protein C anticoagulant pathway. Increased amounts of sTM in the bloodstream indicate damage to endothelial cells (22). Other endothelial and tissue damage biomarkers, including sVEGFr1 (soluble vascular endothelial growth factor receptor 1) and histone-complexed DNA fragments (hcDNA), have been associated with endothelial cell injury and/or dysfunction and are correlated with inflammation, coagulopathy, thrombosis, organ dysfunction, and clinical outcomes in severely injured patients (25, 27, 28).

Trauma also results in coagulation cascade dysfunction that leads to coagulopathy. Acute trauma-induced coagulopathy (TIC) can be manifested as a spectrum of phenotypes from hypocoagulation (characterized by hyperfibrinolysis, platelet dysfunction, fibrinogen depletion, and decreased thrombin generation) to hypercoagulation (defined by increased thrombin generation, hyperfibrinogenemia, platelet activation, and fibrinolysis shutdown) (29). Early TIC (generally within 6 hours post-trauma) is initiated by shock-associated hypoperfusion and IRI, and represented by hypocoagulability resulting in bleeding. In contrast, late TIC (usually >24hours after injury) is triggered by tissue injury and hemodilution, and features a hypercoagulable status associated with thrombosis and MOF (29–31). Unsurprisingly, many of these phenomena are associated with or successfully predict poor clinical outcomes (32–35).

Accumulating evidence suggesting that the complement mediates immune dysfunction in the early phase of trauma might largely depend on its interactions with the coagulation and fibrinolysis pathways and its interactions with endothelial damage factors (2, 15, 36, 37). However, the connections between complementopathy, endotheliopathy and coagulopathy, and their impact on clinical outcomes in trauma have not been fully elucidated. Significant inter-patient heterogeneity of pathological host responses to trauma (10, 16, 25, 27, 38–42) shifted trauma care from a traditional phenotype-driven “one-size-fits-all” management to a rapidly developing paradigm of endotype-driven personalized management. Thus, there is increasing interest in better understanding their interaction and identifying subtypes defined by a distinct pathobiological/functional mechanism based on complementopathy, endotheliopathy, and coagulopathy among individual trauma patients to facilitate precision diagnosis and treatment (38, 43). In this study, we plan to characterize endotypes defined by the complement system, endothelial, and coagulation dysfunction, analyze their relationships and evaluate their association with clinical trajectory and outcomes, focusing on MOF, mortality, and infectious complications in a poly-trauma civilian cohort.

## Materials and methods

### Patients and controls

The University of Texas Health Science Center at Houston (UTHealth Houston) Institutional Review Board (HSC-MS-07-0499) approved this prospective observational study. Adult patients with acute injury admitted to the Emergency Department (ED) of UTHealth Houston, a level 1 trauma center, were included in the study. Exclusion criteria for the study were: pregnancy, prisoners, enrollment into other studies, declined consent, or if no blood samples were drawn on admission. Fifty-four poly-trauma patients were enrolled in this study; blood samples were collected upon admission to the ED and at certain intervals (3h, 6h, 12h, 24h, and 120h) after admission. Samples were collected in citrated tubes and then centrifuged within 2h at 2000 × g for 20 minutes at room temperature. Post-centrifugation, blood plasma was aliquoted, snap-frozen, transported on dry ice to the US Army Institute of Surgical Research (USAISR), and stored at -80°Cfor subsequent analyses. Upon admission to the ED, clinical and demographic characteristics of patients were recorded, including age, gender, race/ethnicity, ISS, pulse rate, systolic blood pressure (SBP), heart rate, base excess/base deficit (BE/BD), and level of consciousness (Glasgow Coma Scale, GCS). Patients were classified as having severe TBI if their Abbreviated Injury Scale (AIS) head/neck score (AIS_brain_) was higher than 2, and there was radiological confirmation of TBI with computed tomography. Transfusion of red blood cell (RBC) units, plasma, platelets, and all blood products, complications of hyperglycemia, bacteremia, respiratory failure, pneumonia, acute kidney injury (AKI), and presence of an asymptomatic urinary tract infection (aUTI) were also recorded, along with the duration of mechanical ventilation, number of intensive care unit (ICU) and hospital stay days, and mortality during hospitalization.

In addition, we enrolled sixteen healthy volunteers at the USAISR as healthy controls. Volunteers were 18 years old or older with no significant medical conditions. Citrated blood samples were withdrawn once and handled in the same way as the trauma patient samples for the analysis of relevant parameters.

### Measurement of complement factors in blood plasma

Quantitative levels of complement factors in human plasma, including C3a, C5a, C5b-9, Bb, and C4d, were measured using commercial enzyme-linked immunosorbent assay (ELISA) kits according to the manufacturer’s instructions (Quidel, San Diego, CA).

### Measurement of endothelial and tissue injury biomarkers in blood plasma

Endothelial damage/dysfunction biomarkers in the plasma, including syndecan-1, sTM, sVEGFr1, and hcDNA, were measured using commercially available ELISA kits according to the manufacturer’s instructions. The detection kits of syndecan-1 and sTM were purchased from Diaclone SAS (Besancon, France) and Nordic Biosite (Copenhagen, Denmark), respectively. ELISA kits for sVEGFr1 and hcDNA were bought from R&D Systems Europe, Ltd. (Abingdon, UK), and Cell Death Detection ELISA^PLUS^ (Roche, Hvidovre, Denmark), respectively.

### Measurement of coagulation parameters in trauma patients

Key coagulation factors (such as V, VIII, and IX) were assessed using chromogenic assays, and plasma levels of factor I, D-dimer, and protein C of the poly-trauma patients were measured using commercially available ELISA kits. The detection kits of factor I and D-dimer were purchased from Abcam (Abcam, Cambridge, MA), and the ELISA kit for protein C was purchased from Helena Laboratories (Beaumont, TX, US), respectively. Thrombin generation of the endogenous thrombin potential (ETP) and the highest amount of thrombin generated (peak) was measured using a calibrated automated thrombogram (Thermo Fisher Scientific, Waltham, MA) as previously described (44). The fibrinolysis parameters of *k*-time, alpha (α)-angle, and percentage of clot lysis at 30 minutes after maximal clot strength (LY30) were tested in the hospital lab by Thrombelastograph 5000 (Haemoscope Corporation, Niles, IL).

### Statistical analysis

Descriptive statistics characterized the demographics and injuries of the patients with *a priori* categorization as mild-, moderate- and severe-injury defined by ISS<16, 16-24, and >24, respectively (45–47). The outcomes of interest were defined as MOF/Death (MOF or death) and infectious complications, including bacteremia, pneumonia, sepsis, and septic shock. Categorical variables were summarized as frequencies and percentages and tested using a Chi-square or Fisher’s exact test for associations between patient characteristics and the three injury severity levels (ISS groups) or a Cochran-Armitage test for trend to assess the presence of binary outcomes of interest and an ordinal variable of injury severity levels. Continuous variables were tested for normality using a combination of descriptive plots (i.e., histogram and Q-Q plots) and the Shapiro-Wilk test. Lavene’s test was used to assess the homogeneity of variance. Normality distributed continuous data were presented as mean and SD or SEM and tested using one-way ANOVA followed by Dunnett’s *post-hoc* test; otherwise, non-normally distributed data were presented as medians with inter-quartile ranges (IQR) and tested using the Mann-Whitney U or Kruskal-Wallis test followed by Dunn’s *post-hoc* test. Longitudinal data were presented as mean and SEM. Since missing data points were encountered in the longitudinal data, a mixed-effect model for repeated measures or Friedman test followed by Dunnett’s or Dunn’s *post-hoc* test where appropriate was used to examine group-specific differences in the least square means of the defined complement, endothelial, and coagulopathy biomarkers throughout the observation period after admission, and at each time point by the injury severity levels as fixed effects. Mauchly’s test for sphericity assumption and the Hotelling-Lawley-McKeon and Hotelling-Lawley-Pillai-Samson for better estimating the degrees of freedom were used where appropriate. An appropriate covariance structure was selected based on the lowest Akaike Information Criterion and Bayesian Information Criterion from a comparison of covariance structures (i.e., unstructured, compound symmetry, Autoregressive, and Huynh-Feldt). Correlations were investigated using Spearman correlations and presented by correlation coefficient (r_s_) and *p*-values. The receiver operator characteristic (ROC) curves were plotted for complement factors (Bb, C3a, and C5b-9), endothelial biomarkers (syndecan-1 and sTM), and coagulation parameters (D-dimer, PTT, PT, and LY30) for predicting MOF/death, or infectious complications in trauma patients. The optimal cut-off values with the Youden index and the areas under the ROC curves (AUROC) were calculated. Sensitivity and specificity using the optimal cut-off values for predicting outcomes were also performed. Logistic regression analysis was used for calculating odds ratios (ORs) with 95% confidence intervals (95% CI) of clinical outcomes of MOF (including AKI and respiratory failure), or death, as well as the secondary clinical outcome of infectious complications. The triadic score was calculated as a sum of scores of Bb (score =1 if Bb >1.57 µg/mL or =0 otherwise), syndecan-1 (score =1 if syndecan-1 > 66.0 ng/ml or =0 otherwise), and D-dimer (score =1 if D-dimer >6.0 mg/L or =0 otherwise) and used to evaluate the synergistic effect of complementopathy, endotheliopathy, and coagulopathy on MOF/death and infectious complications. Statistical significance was determined at the 2-sided *p* < 0.05. The models were adjusted for covariates but not for multiple comparisons. All statistical analyses were performed using SAS, version 9.4 (SAS Institute Inc., Cary, NC) and GraphPad Prism 9.0 (GraphPad Software. San Diego, CA).

## Results

### Patient characteristics

The fifty-four patients included in this study were described in **Table 1**. Most of the patients were males [n=46 (85.2%)] with a median age of 36 years (IQR, 27-52 years) and a median ISS of 21 (IQR, 14-34), comprising 33.3% (n=18) mild injury, 27.8% (n=15) moderate injury, and 38.9% (n=21) severe injury. No statistically significant differences were observed among these three groups in the age, gender, pulse rate, systolic blood pressure (SBP), shock index, and base excess/base deficit (BE/BD) at admission, except the GCS score. It was significantly lower in severely injured patients [median 4 (IQR: 3-15] than moderately [15 (5-15)] or mildly injured patients [15 (11-15)].

**Table 1:** Demographics and injury characteristics.

Parameters	All patients	ISS <16	ISS 16-24	ISS >24
Patients, No. (%)	54 (100.0)	18 (33.3)	15 (27.8)	21 (38.9)
Age, years	36 (27-52)	31 (26-48)	38 (24-61)	40 (29-55)
Sex, male, No. (%)	46 (85.2)	14 (77.8)	15 (100)	17 (80.9)
Race/Ethnicity, No. (%)				
Caucasian	31 (57.4)	9 (50.0)	9 (60.0)	13 (61.9)
Black	12 (22.2)	5 (27.8)	5 (33.3)	2 (9.5)
Others	11 (20.4)	4 (22.2)	1 (6.7)	6 (28.6)
Injury severity score (ISS)	21 (14-34)	12 (9-14)	18 (17-22)	34 (29-41)*†^#^
Pulse rate (bpm)	105 (86-135)	121 (80-145)	100 (88-135)	105 (81-129)
SBP (mmHg)	92 (81-113)	102 (88-112)	97 (84-120)	90 (72-112)
Shock Index	1.1 (0.8-1.4)	1.2 (0.8-1.4)	1.0 (0.8-1.4)	1.1 (0.8-1.4)
BE/BD (mmol/L)	-6.0 (-8.3 - -2.0)	-5.5 (-11.0 - -2.5)	-3.0 (-7.0 - -1.0)	-6.0 (-10.0 - -2.0)
GCS	14 (3-15)	15 (11-15)	15 (5-15)	4 (3-15)*†
RBC_4hrs (units)	5.5 (3.0-10.0)	4.0 (2.8-6.3)	6.0 (3.0-10.0)	6.0 (3.0-11.0)
Plasma_4hrs (units)	5.5 (3.0-10.0)	4.5 (2.0-6.0)	6.0 (2.0-10.0)	6.0 (4.0-10.5)
PLT_4hrs(units)	6.0 (0-6.0)	4.0 (0-6.0)	6.0 (0-6.0)	6.0 (0-12.0)
TBI, yes, No. (%)	15 (27.8)	1 (5.6)	2 (13.3)	12 (57.1)^§^
AIS_brain_ > 2	13 (24.1)	0 (0.00)	1 (6.7)	12 (57.1)^§^
AIS_brain_ 2	2 (3.7)	1 (5.6)	1 (6.7)	0 (0)
AIS_brain_ 3	7 (13.0)	0 (0)	1 (6.7)	6 (50.0)*
AIS_brain_ 4	2 (3.7)	0 (0)	0 (0)	2 (16.7)
AIS_brain_ 5	4 (7.4)	0 (0)	0 (0)	4 (33.3)
Hyperglycemia, No. (%)	30 (55.6)	7 (38.9)	6 (40.0)	17 (81.0)^§^
Bacteremia, No. (%)	11 (20.4)	2 (11.1)	0 (0)	9 (60.0)^§^
Respiratory failure, No. (%)	24 (44.4)	4 (22.2)	5 (33.3)	15 (71.4)^§^
Pneumonia, No. (%)	9 (16.7)	1 (5.6)	0 (0)	8 (38.1)^§^
AKI, No. (%)	14 (25.9)	3 (16.7)	4 (26.7)	7 (33.3)
aUTI, No. (%)	7 (13.0)	2 (11.1)	0 (0)	5 (23.8)
Duration of MV (days)	1 (0-5.0)	0 (0-1.0)	1 (0-5.0)	4 (1-15)*
Duration of ICU (days)	4.0 (1-9.5)	2.0 (0-5.3)	4.0 (1.0-7.0)	9.0 (2.0-18.5)*†
Duration of Hospitalization (days)	14.0 (6.5-24.0)	8.0(4.5-17.0)	12.0 (5.0-19.0)	18.0 (9.5-28.5)*
Death, No. (%)	4 (7.4)	1 (5.6)	0 (0)	3 (14.3)
MOF/Death, No. (%)	29 (53.7)	6 (33.3)	7 (46.7)	16 (76.2)^§^

AIS, abbreviated injury scale; SBP, systolic blood pressure; BE/BD, base excess/base deficit; GCS, Glasgow Coma Scale; RBC, red blood cell; PLT, platelet; AKI, acute kidney injury; aUTI, asymptomatic urinary tract infection, MV, mechanical ventilation, ICU, intensive care unit. Data are presented as median (interquartile ranges, IQR) or otherwise indicated. Statistical analyses were analyzed by using the Kruskal-Wallis test followed by the Dunn’s post hoc test for continuous variables and using Fisher’s exact test or Cochran-Armitage test for trend for ordinal variables where appropriate. *p<0.05, ISS>24 vs. ISS<16; ^†^p<0.05, ISS>24 vs. ISS=16-24; ^#^p<0.05, ISS=16-24 vs. ISS<16; and ^§^ P-trend <0.05 from the Cochran-Armitage trend test.

Four hours after admission, the transfusion units of red blood cells (RBCs), plasma, and platelets were not statistically different between the three groups. Comparative analyses showed a higher risk of developing complications in those with severe injury compared to those with moderate or mild injury, including hyperglycemia (81.0% vs. 40.0% vs. 38.9%), bacteremia (60.0% vs. 0.0% vs. 11.1%), respiratory failure (71.4% vs. 33.3% vs. 22.2%) and pneumonia (38.1% vs. 0.0% vs. 5.6%, respectively). Severe injury (ISS>24) was associated with longer days on mechanical ventilation (4 vs. 0 days) and extended ICU (9 vs. 2 days) and hospital stays (18 vs. 8 days) when compared to mildly injured patients (ISS<16). Overall, 27.8% (n=15) of patients sustained TBI with a higher frequency in the severe injury group (57.1% vs. 13.3% vs 5.6%), and proportion of severe TBI defined as AIS_brain_ > 2 was higher in the severely injured patients compared to moderate and mild group (57.1% vs 6.7% vs 0.0%, respectively) (**Table 1**). Overall mortality was 7.4% (n=4) and was higher in severely injured patients (14.3%) but did not reach a statistically significant difference among the groups in this cohort. However, the percentage of patients who experienced MOF or death combined into one category was associated with the increasing increments of injury severity levels, 33.3% vs. 46.7% vs. 76.2%; p-trend=0.0069 (**Table 1**).

### Complement activation and its relation to injury severity level

**Figure 1:**
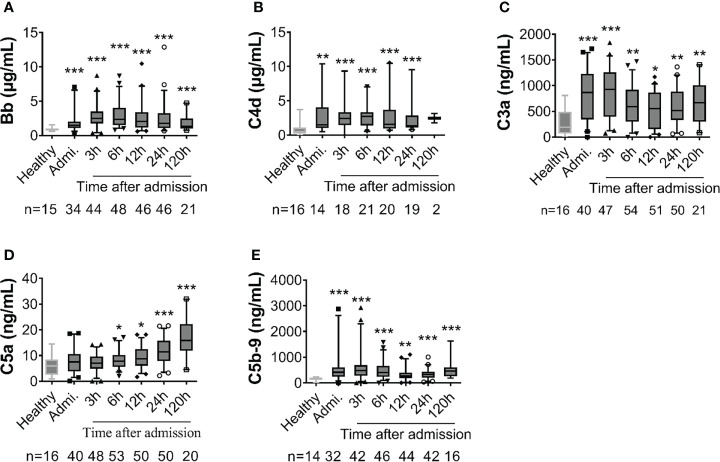
Early complement activation is detected in poly-trauma patients. The plasma samples were collected from patients on admission and at 3h, 6h, 12h, 24h, and 120h after admission, and the complement factors including Bb **(A)**, C4d **(B)**, C3a **(C)**, C5a **(D)** and C5b-9 **(E)** were measured by ELISA. Healthy volunteers were also measured as a control. The numbers of analyzed patients for the individual groups are displayed in each column. The data were presented as mean ± SEM. T-test or Mann-Whitney U test was used to compare f differences between injured patients at the defined time point and the healthy control group. * p<0.05, ** p<0.01, *** p<0.001.

**Figure 2:**
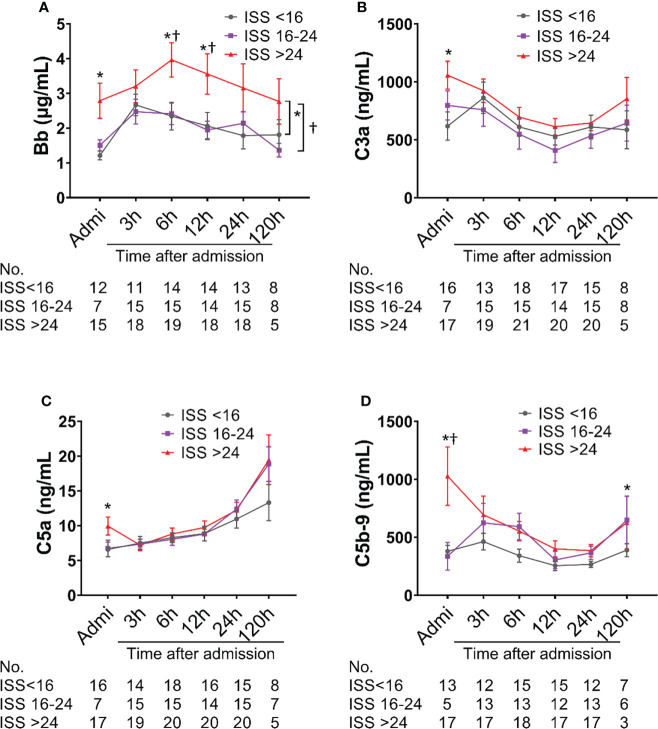
Complement activation is associated with injury severity scores (ISS) in poly-trauma patients. The poly-trauma patients were divided into three groups ISS<16, ISS=16-24, and ISS>24, and complement factors Bb **(A)**, C3a **(B)**, C5a **(C)**, and C5b-9 **(D)** in different categories were calculated, and the data were presented as mean ± SEM. The numbers of analyzed patients for the individual groups are displayed below the graph on each panel. Statistical analyses were performed by linear mixed-effect model for repeated measures and the least square means of each group comparison. *p<0.05, ISS>24 vs. ISS<16; †p<0.05, ISS>24 vs. ISS=16-24.

**Figure 3:**
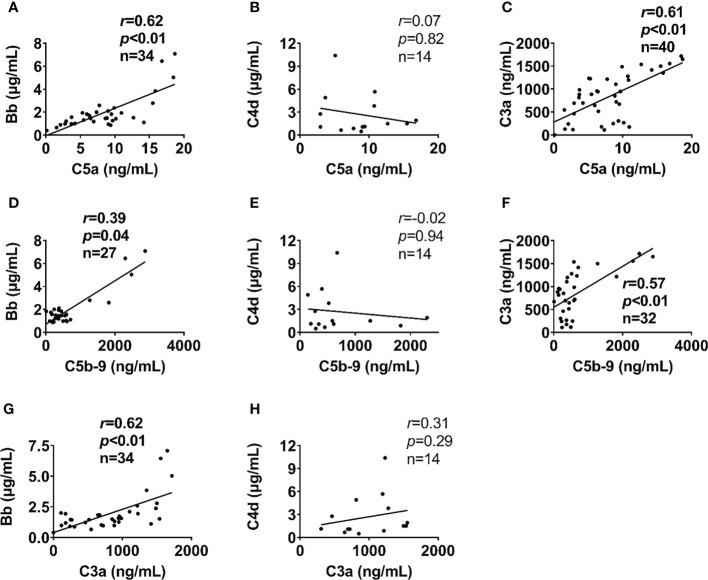
Alternative complement pathway correlates with terminal complement pathway in poly-trauma patients. The alternative complement pathway component Bb on admission **(A, D)**, but not the classic/lectin complement pathway factor C4d **(B, E)** correlated with admission plasma levels of terminal complement factors (C5a and C5b-9). In addition, the complement factor C3a on admission **(C, F)** correlated with admission plasma levels of terminal complement pathway factors (C5a and C5b-9). Bb **(G)** but not C4d **(H)** associated with C3a. The correlation analyses were performed using Spearman’s rank correlation; the data were presented with a coefficient (r_s_) and *p* values. Significant correlations (*p <*0.05) are indicated by boldface type.

Complement factors were significantly elevated after traumatic injury compared with healthy controls: The level of complement factors Bb, C4d, C3a, and C5b-9 in the blood plasma significantly increased on admission and remained high throughout the observation period (**Figure 1**). However, the level of complement factor C5a gradually increased after admission through 24h and 120h (**Figure 1D**), while other complement factors gradually declined or remained stable (**Figures 1A–C, E**). Comparative analyses showed that these complement factors were significantly higher in severely injured patients at admission and during follow-up than in less severely injured patients (**Figure 2**). The level of complement factor Bb at 6h and 12h post-admission and the least square means were significantly higher in severely injured patients (ISS>24) compared to those with mild (ISS<16) and moderate injury (ISS=16-24) (**Figure 2A**), while C3a, C5a, and C5b-9 were no differences in the least square means. Correlation analyses also revealed positive correlations between the amount of Bb and C5a, C5b-9, and C3a on admission (**Figures 3A, D, G**) and between the amount of C3a with C5a and C5b-9 (**Figures 3C, F**). We observed no correlation between levels of C4d with C5a, C5b-9, and C3a (**Figures 3B, E, H**).

### Endothelial damage in polytrauma patients

**Figure 4:**
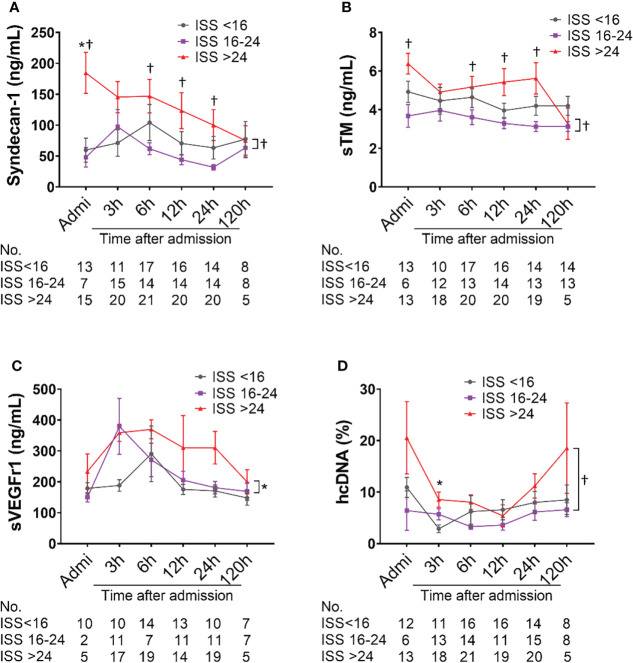
Biomarkers of endothelial cell and tissue damage are elevated in poly-trauma patients. The blood samples were collected upon admission, and at 3h, 6h, 12h, 24h, and 120h after admission, and the endothelial cell and tissue damage biomarkers of Syndecan-1 **(A)**, sTM **(B)**, sVEGFr1 **(C)**, and hcDNA **(D)** were measured by ELISA. The numbers of analyzed patients for the individual groups are displayed below the graph on each panel. The data were presented as mean ± SEM. Statistical analyses were performed by linear mixed-effect model for repeated measures and the least square means of each group comparison. * p<0.05, ISS>24 vs. ISS<16; † *p*<0.05, ISS>24 vs. ISS=16-24.

The amount of syndecan-1 and sTM were higher in severely injured patients (ISS>24) compared with moderately injured patients (ISS=16-24) at admission, 6h, and 12h after admission; there were significant differences in the least square means between severely and mildly injured patients (p=0.0241 and p=0.0084, respectively) (**Figures 4A, B**). A significant increase in the least square means of sVEGFr1 (p=0.0429) and hcDNA (p=0.0352) was also seen in severely injured patients compared to mildly and moderately injured patients, respectively (**Figures 4C, D**).

### Coagulopathy in poly-trauma patients

**Figure 5:**
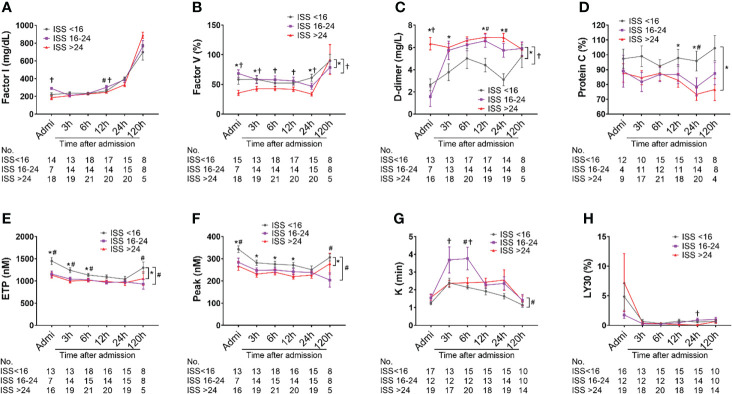
Coagulation and fibrinolysis parameter abnormalities in poly-trauma patients. The blood samples were collected upon admission, and at 3h, 6h, 12h, 24h, and 120h after admission, and the coagulation and fibrinolysis parameters of factor I **(A)**, factor V **(B)**, D-dimer **(C)**, sCD40L **(D)**, ETP **(E)**, peak **(F)**, *k*-time **(G)**, and LY30 **(H)** were measured by chromogenic assays, ELISA or TEG machines. The coagulation parameters in three different groups as ISS<16, ISS=16-24, and ISS>24 were calculated and the data were presented as mean ± SEM. The numbers of analyzed patients for the individual groups are displayed below the graph on each panel. Statistical analyses were performed by linear mixed-effect model for repeated measures and the least square means of each group comparison. * p<0.05, ISS>24 vs. ISS<16; † *p*<0.05, ISS>25 vs. ISS=16-24; # p<0.05, ISS=16-24 vs. ISS<16.

Factor I concentration in patients with ISS>24 was significantly lower on admission compared with less severely injured patients (ISS=16-24 & <16), but no differences in the least square means between the ISS levels were observed (**Figure 5A**). Factor V level in patients with ISS>24 was significantly lower than in those patients with ISS=16-24 and ISS<16 on admission, at 3h, 6h, 12h and 24h after admission. There were significant differences in the least square means in severely injured patients compared to mildly and moderately injured patients (**Figure 5B**). A significantly higher level of D-dimer and significant differences in the least square means were observed in severely injured patients at admission through 120h than in less severely injured patients (**Figure 5C**). The level of protein C was found to be significantly lower in severely injured patients than in less injured patients at 12h and 24h post-admission; there was a significant difference in overall least square mean between severely and less injured patients (**Figure 5D**). The overall thrombin generation was deceased after traumatic injury, which was characterized by significantly decreased ETP and peak values in moderately and severely injured patients compared to less injured patients at multiple time points, including admission, 3h, 12h, and 120 h after admission; significant differences in the least square means were detected in moderately and severely injured patients compared to mildly injured patients (**Figures 5E, F**). The fibrinolysis parameter of *K*-time was significantly increased in moderately injured patients compared to mildly injured patients, but significantly decreased in severely injured patients at 3h and 6h after admission. The difference in the least square means was noticed between moderate and mild injured patients (**Figure 5G**). LY30, a parameter representing fibrinolysis and clot breakdown, had no significant differences in severely injured patients when compared with less injured patients (**Figure 5H**). We also showed lower concentrations of Factors VIII and IX, and lower angle values seemed to be in more severely injured patients than less injured patients (**Supplemental Figure 1**).

### Interaction of complement, endothelial, fibrinolysis and coagulation systems

**Figure 6:**
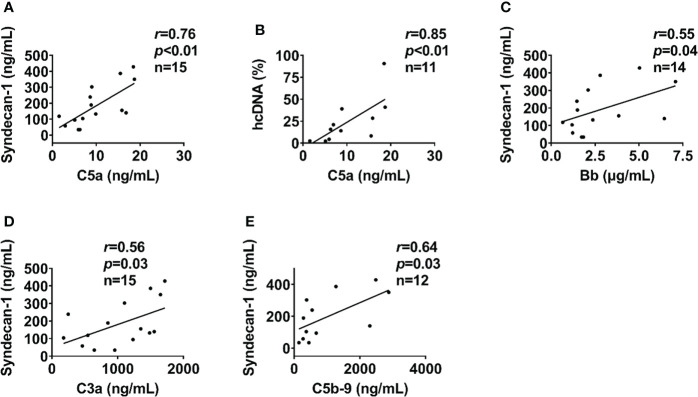
Endothelial cell and tissue damage biomarkers lcorrelate with complement in severely injured patients. In severely injured patients (ISS>24), the endothelial cell and tissue damage biomarkers of syndecan-1 and hcDNA correlated with complement factors of C5a at admission **(A, B)**. In addition, syndecan-1 also correlated to Bb **(C)**, C3a **(D)**, and C5b-9 **(E)** in severely injured patients (ISS>24) at admission. The correlation analyses were performed using Spearman’s rank correlation; the data were presented with a coefficient (r_s_) and *p* values. Significant correlations (*p <*0.05) are indicated by boldface type.

**Figure 7:**
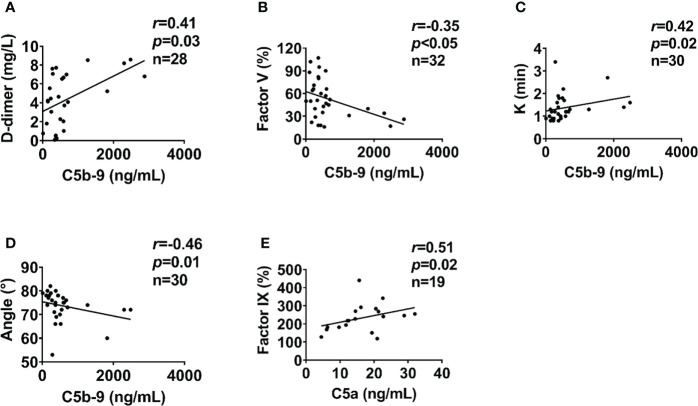
Coagulation parameters correlate with complement levels in poly-trauma patients. At admission, the coagulation parameters D-dimer **(A)**, factor V **(B)**, k-time **(C)**, and Angle **(D)** correlated with complement factor C5b-9. At 120h post-admission coagulation factor IX correlated with complement factor C5a **(E)**. The correlation analyses were performed using Spearman’s rank correlation; the data were presented with a coefficient (r_s_) and *p* values. Significant correlations (*p <*0.05) are indicated by boldface type.

In severely injured patients (ISS>24), complement activation positively correlated with biomarkers of endothelial dysfunction. C5a level positively correlated with syndecan-1 and hcDNA levels (**Figures 6A, B**). In addition, syndecan-1 level positively correlated with Bb, C3a, and C5b-9 levels in patients with ISS>24 at admission (**Figures 6C–E**). These complement factors also correlated with the factors of coagulation and fibrinolysis pathways in this trauma cohort. C5b-9 level positively correlated with D-dimer level and *k*-time as detected at admission (**Figures 7A, C**), but negatively correlated with factor V concentration and angle (**Figures 7B, D**). There was a positive correlation between C5a and factor IX at 120h after admission (**Figure 7E**). Interestingly, endothelial damage biomarkers syndecan-1 and sTM highly correlated with changes in multiple coagulation and fibrinolysis parameters, such as factor I, factor V, D-dimer, peak, *k*-time, and factor IX (**Table 2**).

**Table 2:** Endothelial damages correlate with the changes of coagulation parameters in poly-trauma patients.

Parameters		Syndecan-1	sTM
Factor I	*r*	-0.41	-0.43
	*p*	0.02	0.01
Factor V	*r*	-0.59	-0.27
	*p*	<0.01	0.14
D-Dimer	*r*	0.61	0.49
	*p*	<0.01	<0.01
Protein C	*r*	-0.24	0.14
	*p*	0.26	0.53
ETP	*R*	-0.15	-0.07
	*p*	0.41	0.71
Peak	*r*	-0.36	-0.26
	*p*	0.04	0.17
K	*r*	0.36	0.11
	*p*	0.04	0.57
LY30	*r*	0.04	0
	*p*	0.84	0.99
Factor VIII	*r*	-0.22	-0.22
	*p*	0.2	0.21
Factor IX	*r*	-0.42	-0.25
	*p*	0.01	0.17
Angle	*r*	-0.32	-0.02
	*p*	0.07	0.92

ETP, endogenous thrombin potential; LY30, clot lysis at 30 minutes after maximum clot strength; sTM, soluble thrombomodulin. The correlation analyses were performed by using Spearman’s rank correlation, the data were presented with a coefficient (r_s_) and p values.

### Biomarkers factor Bb, syndecan-1, and D-dimer as predictors of MOF/death, and infectious complications

**Figure 8:**
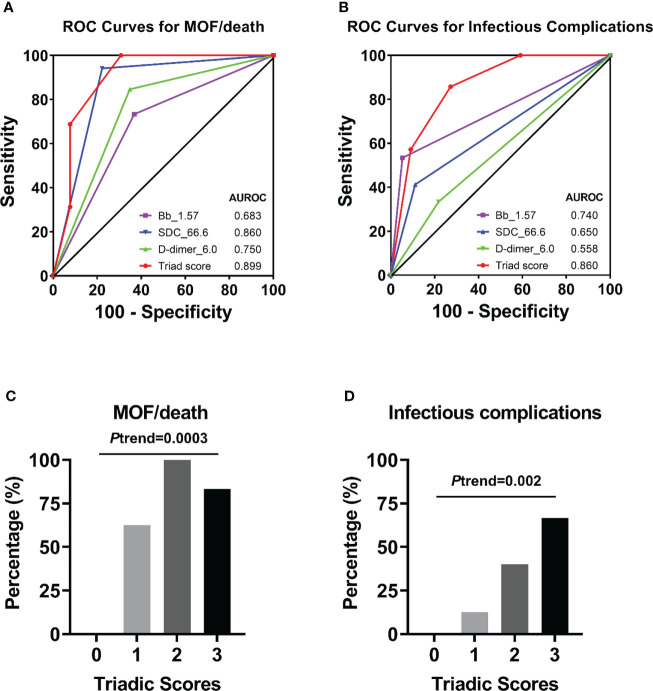
Receiver operator characteristic (ROC) curves of an individual biomarker or triadic score for predicting clinical complications. ROC curves plotted for studying of triadic score or individual biomarker Bb (with cut-off=1.57 µg/mL), syndecan-1 (with cut-off=66.6 ng/mL), D-dimer (with cut-off=6.0 mg/L) in predicting of MOF/death **(A)**, or infectious complications **(B)**. The percentage of patients with MOF/death **(C)** or infectious complications **(D)** associated with the triadic scores was also presented.

ROC analyses revealed that complement factor Bb, endothelial biomarker syndecan-1, and coagulation parameter D-dimer at admission were the representative factors in each pathway potentially associated with clinical outcomes of MOF/death (**Supplemental Figure 2**). The cut-off value of each of the above biomarkers that best predicts the clinical outcome of MOF/death was further assessed (**Supplemental Figure 3**), and the value for Bb, syndecan-1, and D-dimer were presented as 1.57 µg/mL, 66.6 ng/mL, and 6.0 mg/L, respectively. The concentration of these three biomarkers determined the triadic score, as described in the methods section. In terms of the triadic score’s relationship to clinical outcome, the AUROC data showed that the triadic score, which represents a synergy of complementopathy, endotheliopathy, and coagulopathy, is a better predictor of MOF/death and infectious complications than analysis of the above biomarkers separately (**Figures 8A, B**). Strikingly, the increasing triadic score was also significantly associated with incremental proportions of MOF/death and infectious complications (**Figures 8C, D**).

### Association of the triadic score and MOF/death and infectious complications

Logistic regression analyses demonstrated that the triadic score is significantly associated with clinical outcomes (**Table 3**). Individuals with Bb>1.57 μg/mL were associated with a 3-time [OR=3.75 (0.87-16.22); *p*=0.077] or a 25-time [OR=25.33 (2.61-245.73); *p*=0.005] higher risk of MOF/death or infectious complications respectively and individuals with syndecan-1>66.6 ng/mL were associated with a 56-time [OR=56 (5.58-561.75); *p*=0.001] or a 5-time [OR=5.6(0.96-32.51); *p*=0.055] higher risk of MOF/death or infectious complications respectively. While coagulation parameter D-dimer was associated with a 10-time [OR=10.31 (1.82-58.37); *p*=0.008] higher risk of MOF/death, it was not statistically identified as indicative of infectious complications. Interestingly, logistic regression analyses using a panel of all three pathways combined into the triadic score (Bb>1.57 μg/mL, syndecan-1>66.6 ng/ml, and D-dimer>6.0 mg/L) revealed that each increase in triadic score was associated with a 7-time [OR=7.39(1.86-29.42); *p*=0.005] higher in odds of MOF/death. After adjusting for ISS, each increased point of the triadic score was associated with a 6-time greater in odds of MOF/death [OR=6.83 (1.04-44.96); *p*=0.046] and a 4-time higher in odds of infectious complication [OR=4.12 (1.04-16.36); p=0.044] (**Table 3**).

**Table 3:** Factors Associated with Odds of MOF/death and Infectious Complications.

Univariate	MOF/death		Infectious Complications	
	OR (95% CI)	*P* value	OR (95% CI)	*P* value
Gender, No. (%)				
Male vs. Female	2.17 (0.46-10.16)	0.327	2.20 (0.24-19.90)	0.483
Age at injury, each age increase	1.01 (0.98-1.05)	0.445	1.01 (0.97-1.04)	0.742
Race/Ethnicity				
Black vs. Caucasian	0.52 (0.13-1.99)	0.337	n/a	n/a
Other vs. Caucasian	0.87 (0.22-3.46)	0.840	5.00 (1.13-22.05)	0.034
Other vs. Black	1.68 (0.32-8.76)	0.538	n/a	n/a
ISS, each unit increase	1.11 (1.04-1.18)	0.002	1.09 (1.03-1.16)	0.006
Type of injury				
Blunt vs. penetrating	3.54 (1.07-11.66)	0.038	7.48 (0.88-63.44)	0.065
TBI, Yes vs. No	3.21 (0.87-11.84)	0.080	3.67 (0.95-14.15)	0.059
Complementopathy				
C3a	1.00 (0.99-1.00)	0.479	1.00 (0.99-1.00)	0.233
C5b-9	1.00 (0.99-1.00)	0.567	1.00 (1.00-1.00)	0.241
Bb	1.34 (0.78-2.31)	0.289	1.44 (0.88-2.36)	0.146
Bb ≥1.57 vs. <1.57	3.75 (0.87-16.22)	0.077	25.33 (2.61-245.73)	0.005
Endotheliopathy				
sTM	1.30 (0.90-1.87)	0.165	1.12 (0.74-1.68)	0.604
Syndecan-1	1.01 (1.00-1.03)	0.034	1.01 (1.00-1.02)	0.034
Syndecan-1, ≥66.6 vs. <66.6	56.00 (5.58-561.75)	0.001	5.60 (0.96-32.51)	0.055
Coagulopathy				
PT	1.00 (0.98-1.03)	0.799	1.01 (0.98-1.04)	0.459
PTT	1.12 (0.98-1.29)	0.088	1.01 (0.99-1.03)	0.266
LY30	1.03 (0.96-1.11)	0.385	1.02 (0.98-1.07)	0.316
D-dimer	1.22 (0.95-1.55)	0.117	14.67 (1.73-124.20)	0.014
D-dimer, ≥6.0 vs. <6.0	10.31 (1.82-58.37)	0.008	1.60 (0.34-7.46)	0.549
Triadic score*, each score increase	7.39 (1.86-29.42)	0.005	4.65 (1.41-15.30)	0.012
Adjusted models				
Triadic score, each score increase. Adjusted for ISS	6.83 (1.04-44.96)	0.046	4.12 (1.04-16.36)	0.044

PT, prothrombin time; PTT, Partial thromboplastin time; LY30, percentage of clot lysis at 30 min after maximal clot strength;MOF, Multiple organ failure. *, Triadic score was caculated as a sum of scores of Bb (score=1 if Bb>1.57 µg/mL or=0 otherwise), syndecan-1 (score=1 if syndecan-1>66.6 ng/mL or=0 otherwise) and D-dimer (score=1 if D-dimer ≥6.0 or=0 otherwise). P-values were calculated using the logistic regression. n/a, not applicable. n/a, not applicable.

## Discussion

In this study, we have shown that (1) early complement activation correlates with endotheliopathy and coagulopathy after trauma; (2) plasma levels of Bb>1.57 μg/ml, syndecan-1>66.6 ng/ml, and D-dimer >6.0 mg/L are linked to a higher risk of MOF/death; and (3) each increased score of the triadic score is associated with a higher risk of MOF/death and infectious complication. Altogether, these data highlight that the triad of complementopathy, endotheliopathy, and coagulopathy may represent a potential endotype of trauma patients, and serve as a distinguishing prognostic/diagnostic indicator and a potential therapeutic target for clinical trauma care.

It is well known that severe trauma triggers a profound immune response characterized by systemic inflammation, cytokine storm, complement activation, and coagulopathy (14, 20). These events lead to endothelial damage, SIRS, CARS, and/or PICS, subsequently causing MOF and significant mortality (17–20). Complement, a key innate immune mediator, was typically detected early after traumatic injury. Complement activation can be driven by DAMPs/PAMPs and post-trauma early therapeutic approaches (15). Abnormal activation of complement can lead to complementopathy, which is considered to contribute to high morbidity and mortality (10, 16). In severe trauma patients, complementopathy is a predominant phenomenon occurring early after injury (10). Burk AM et al. found that within a poly-trauma population (mean ISS=30.3 ± 2.9), the complement activation, assessed by analyzing CH50, was massively reduced at 4h after trauma, gradually increased to the level of activity observed in healthy volunteers by 5 days, then significantly increased throughout a period of ten days after trauma (10). Ganter MT et al. found that the complement was activated 30 minutes after injury, even before any significant fluid resuscitation, and both C5b-9 and Bb were associated with high ISS (39). In our recent work (16), we analyzed 21 trauma patients (with a mean ISS=25) and found that the soluble C5b-9 was significantly increased in trauma patients over seven days after hospital admission.

As an immune regulator, the complement system closely interacts with other important systems, including the coagulation system (15, 48) and adaptive immunity (14). Mounting evidence suggests that trauma-induced complement activation may provoke endotheliopathy that leads to SIRS, vascular hyperpermeability, MOF, and thrombotic thrombocytopenic purpura *via* the “two-path unifying theory”: (1) C5b-9-endotheliopathy-cytokine storm and (2) C5b-9-endotheliopathy-platelet activation/endothelial exocytosis of unusually large von Willebrand factor (49, 50). In line with this, this study suggests that complement might also tightly regulate endotheliopathy. “Endotheliopathy of trauma” is a term first proposed by Holcomb and Pati (51), who attempted to describe a syndrome involving the early breakdown of the endothelial glycocalyx layer (EGL) after injury. Damage to the EGL increases vascular permeability and causes capillary leakage resulting in the exposure of endothelial cells to circulating platelets and leukocytes. These events instigate the acute inflammatory response, which may alter coagulation (52, 53). The EGL serves as a mechanosensor. It regulates vascular permeability, mediates hemodynamic (mechanical) forces, including shear stress sensing, pressure, and circumferential stretch, and weakens blood cell–vessel wall interaction (54). As a result, systemic EGL breakdown triggers downstream responses, which leads to deleterious systemic effects (i.e., thromboinflammation, edema, organ-barrier dysfunction, and endothelial dysfunction) (25, 53, 55).

Syndecan-1 and sTM are considered well-established biomarkers of endothelial damage. Syndecan-1, the major component of EGL, has been widely studied in traumatic injury and is currently considered a key marker of endothelial glycocalyx degradation (26). The highest syndecan-1 levels in severe trauma patients reflect the highest glycocalyx damage. In one study, there was a several-fold higher mortality rate in those patients when compared to patients with lower syndecan-1 levels (25). Thrombomodulin, an anticoagulant protein expressed on the surface of endothelial cells, plays a major role in the activation of the protein C anticoagulant pathway. Damage to the endothelial cells induces the release of thrombomodulin in its soluble form (sTM). Notably, both sTM and syndecan-1 were correlated with ISS, inflammation (IL-6, hcDNA, HMGB1), and TIC (22, 25, 52). Syndecan-1 and sTM are also strongly associated with organ (liver and renal) failure in critically ill patients with sepsis (56). Quantitative data analysis by Gonzalez Rodriguez et al. showed that patients with a syndecan-1 level higher than 40 ng/mL in the blood plasma had higher 30-day mortality (57). The data from our study agree that poly-traumatized patients have significantly elevated syndecan-1 and sTM levels, which positively correlated with ISS. In our study, we observed that syndecan-1 levels in trauma patients are much higher than 40 ng/mL during the first 120 hours after injury, especially in severely injured patients, wherein the syndecan-1 levels (median) mostly reached 200 ng/mL.

Aside from syndecan-1 and sTM, other endothelial injury-related biomarkers were also analyzed within this trauma cohort. Circulating sVEGFr1 levels positively correlated with ISS, endothelial damage/activation (syndecan-1, sTM, angiopoitin-2), hyperfibrinolysis (tissue plasminogen activator, D-dimer), tissue hypoperfusion, tissue injury (hcDNA), inflammation (IL-6), and transfusion requirements in trauma patients (28, 58) and a preclinical animal model of hemorrhage-induced endotheliopathy (59). Aligned with these earlier reports, our findings also revealed a correlation of sVEGFr1 with ISS. sVEGFr1 was reported to protect against cerebral IRI through attenuation of blood-brain barrier permeability (60). While not as overly pro-angiogenic as VEGFr2, VEGFr1 mRNA and protein were expressed on endothelial cells in and around the lesion, suggesting that it is related to pathological angiogenesis (61–63).

Circulating hcDNA, and protein C (a coagulation inhibitor that also has anti-inflammatory activity (64)), are all deemed to be associated with endothelial damage/dysfunction, inflammation, coagulopathy, and poor clinical outcomes in trauma patients (25, 27, 28, 65). Our data are consistent with previous observations that these biomarkers were significantly increased after severe trauma, and their elevations correlated with ISS. Our study first revealed that the above biomarkers (syndecan-1, hcDNA, and protein C) have a strong association with the alternative/terminal complement activation in severely injured patients. Further and specific studies are required for a deeper understanding of the underlying mechanisms of complement-endothelial cell interactions.

TIC is characterized by early hypocoagulopathy and late hypercoagulopathy (40). In severe trauma injuries, the early hypocoagulopathy is portrayed by hyperfibrinolysis, platelet dysfunction, fibrinogen depletion, and decreased thrombin generation provoked by shock-related hypoperfusion and IRI (29). Our study consistently supports previously reported findings that hypocoagulopathy occurred immediately after trauma. Fibrinolytic dysregulation postinjury can manifest as three phenotypes: hyperfibrinolysis, physiological fibrinolysis, and hypofibrinolysis (shutdown). Hyperfibrinolysis is associated with early mortality because of uncontrolled bleeding, while fibrinolysis shutdown, a predominant clinical phenotype (64%), is associated with late mortality from TBI and/or MOF (66, 67). Hyperfibrinolysis can be induced by tissue plasminogen activator (t-PA) release, due to damage to the endothelium as a result of massive tissue injury, as well as coagulation activation (11, 41). Many severely injured trauma patients with early hypocoagulopathy evidenced by reduced thrombin generation (prothrombin fragment 1 + 2), hyperfibrinolysis [elevated t-PA, reduced plasminogen activation inhibitor-1, and increased D-dimer], endotheliopathy, coagulation factor consumption, and inflammation (41, 42). Aligned with these results, our data showed significantly higher levels of D-dimer and K-time, and lower values of ETP, peak, and angle in patients with moderate/severe trauma on admission. This observation indicates early hyperfibrinolysis occurring in these trauma patients. In addition, the data presented here show significantly decreased levels of multiple coagulation factors in the patients tested upon admission. In fact, consumption of clotting factors and platelets are typical phenomena consistently reported in the acute phase of trauma (68, 69).

The existence of a cross-talk between the complement and coagulation cascades has been broadly discussed. Huber-Lang et al. discovered that thrombin in a proteolytic pattern activates C5 to generate C5a in the absence of C3 (70). Amara et al. found that coagulation/fibrinolysis factors activate complement components C3 and C5, which subsequently activate the complement pathway (37). Similarly, Gulla et al. reported that the complement is a pro-coagulant factor leading to the activation of thrombin, which generates fibrin mesh (71). Importantly, the data reported here add useful information to the field of trauma. Our findings showed that the synergistic effects of complementopathy, endotheliopathy, and coagulopathy occurred early after trauma, contributed to poor clinical outcomes (MOF/death), and led to infectious complications; therefore, the triadic intercommunication model is proposed. Additionally, biomarkers such as Bb, syndecan-1, and D-dimer are reliable early predictive biomarkers of clinical outcomes.

This poly-trauma cohort showed that syndecan-1 levels at an optimal cut-off value ≥66.6 ng/mL could predict MOF/death. This cut-off value is slightly higher compared with those previously reported. Keyloun J et al. found that the syndecan-1 levels at >34 ng/mL were associated with a higher risk of 30-day mortality in burned patients (72). Rodriguez EG et al. defined syndecan-1 level at >40 ng/mL as predicting 24h in-hospital mortality (57). However, Wernly B et al., reported that a syndecan-1 level >120 ng/mL is associated with 6-month mortality in myocardial infarction patients (73). One of the potential explanations is that the syndecan-1 levels varied in different patients because of various types of injury mechanisms. We also determined the admission cut-off values of Bb >1.57 μg/mL and D-dimer>6.0 mg/L as a prognostic factor for MOF/death. Similarly, as reported before D-dimer>2.025 mg/L was regarded to as the optimal probability cutoff for a prognosis of death in COVID patients (74). Taken together, we could analyze three biomarkers, Bb, syndecan-1, and D-dimer as representative of the complement, endothelial, and coagulation pathways in the prognosis of clinical outcomes. Presented data further reinforce the potential of these biomarkers to serve as a potential prognostic/diagnostic tool for trauma patients suffering from MOF/death and infectious complications.

Recent studies suggest that TBI has substantial cofounding and/or modifying effects on risk factors and outcomes of intravascular innate immune system. Primary TBI initiates DAMPs release that subsequently activates microglia/astrocytes and complement cascade, leading to generation of chemokines, cytokines, reactive oxygen species, and excitatory neurotransmitters, and further release of DAMPs, ultimately resulting in secondary brain injury (neuroinflammation, blood brain barrier damage, brain edema, neuronal death, increased intracranial pressure, hypoxia, etc.) (2). Release of brain-derived DAMPs, coagulation factors (t-PA, TF, phospholipids, etc.) and cellular microvesicles along with post-TBI activation of autonomic nervous system and hypothalamic-pituitary-adrenal axis leads to systemic inflammation, complementopathy, endotheliopathy, and TIC that induce remote organ injury (2, 29, 75–77). Our data in this study showed that severely injured patients had a higher incidence of TBI, MOF/death, and other clinical outcomes. Unlike other groups’ reports (10, 11, 76–78), we did not observe a significant correlation between TBI, complementopathy, endotheliopathy, and TIC, most likely due to the small sample size. Further studies need to explore the relationship in larger cohort trauma patients with and without TBI.

Our study has several limitations. First, the data represents a single-institution with a relatively small sample size, especially with only a few fatal cases. We could not identify a statistically significant association between ISS levels or triadic score and the clinical outcome of death. Hence, we combined MOF and mortality as a primary outcome to overcome this limitation. A sample size of 29 patients who had all three biomarkers included to evaluate a viable triadic score and logistic regression for the primary binary outcome of MOF/death on a continuous triadic score variable, achieves a power of ~ 80% and 95% confidence intervals to detect a change in probability of MOF/death. The triadic score is a potential predictor of clinical outcomes, it should be validated with larger cohort studies. Second, compared with EDTA plasma, the citrated plasma could affect the complement biomarker measurement in this study because citrate does not block complement activation effectively (79). Third, the lack of thrombin generation markers (e.g., soluble fibrin, thrombin fragment 1 + 2, thrombin-antithrombin complexes) is another limitation in this current study. Finally, a recent study has reported that there is no difference in overall outcomes between viscoelastic hemostatic assays (VHAs) including TEG and conventional coagulation tests (80). Although the evidence in TIC is limited at present, substantial evidence from elective cardiac and liver transplant surgery studies provides further support for the use of VHAs (81). Nevertheless, VHAs have been adopted for diagnosis of TIC in many countries (82–85) owing to their real-time assessment of whole-blood clot formation and degradation. As the technology advances, ongoing studies should improve the overlying algorithms that VHA-derived data provide us during the management of bleeding trauma patients.

## Conclusion

This exploratory study provides preliminary evidence for a potential endotype among the trauma patients defined by the triad of complementopathy, endotheliopathy, and coagulopathy, which may be uniquely associated with poor clinical outcomes. Therefore, developing plasma-based resuscitation with an appropriate adjunctive pharmacological approach to prevent the early onset of MOF/death may require a combined therapeutic regimen targeting all three aforementioned pathways involved in the early phase care of the traumatic injury. The triadic score may be a potential indicator in trauma that could have utility as a prognostic/diagnostic approach for MOF/death and a stratification tool for enrollment of subjects in clinical therapeutic targeting of the triad. Although the triad score is a potential predictor of clinical outcomes, further analysis from larger datasets with higher mortality rates and higher stratified injury severity is warranted.

## Data availability statement

The original contributions presented in the study are included in the article/**Supplementary Material**. Further inquiries can be directed to the corresponding author.

## Ethics statement

The University of Texas Health Science Center at Houston (UTHealth Houston) Institutional Review Board (HSC-MS-07-0499) approved this prospective observational study. Written informed consent for participation was not required for this study in accordance with the national legislation and the institutional requirements.

## Author contributions

ZY, TL, MS, BL, TF, and YL analyzed the data; ZY, TL, and MS performed statistical analysis; BL and YL performed experiments and measurements; ZY and MS took part in the drafting manuscript. ZY, TL, MS, TC, AC, CW, JD, and YL reviewed and edited the manuscript. CW provided human blood samples. AC, JD, and YL designed the research. All authors contributed to the article and approved the submitted version.

## Funding

The DoD US Army Medical Research & Development Command (C_038_2014) supported this research.

## Acknowledgments

The authors thank all of the research assistants for their work and commitment.

## Conflict of interest

The authors declare that the research was conducted in the absence of any commercial or financial relationships that could be construed as a potential conflict of interest.

## Publisher’s note

All claims expressed in this article are solely those of the authors and do not necessarily represent those of their affiliated organizations, or those of the publisher, the editors and the reviewers. Any product that may be evaluated in this article, or claim that may be made by its manufacturer, is not guaranteed or endorsed by the publisher.

## Author disclaimer

The views expressed in this article are those of the authors and do not reflect the official policy or position of the U.S. Army Medical Department, Department of the Army, DoD, or the U.S. Government.

## Supplementary material

Supplementary Figure 1 Coagulation and fibrinolysis parameters abnormalities in poly-trauma patients. The blood samples were collected upon admission and at 3h, 6h, 12h, 24h, and 120h after admission, and the coagulation and fibrinolysis parameters of factor VIII **(A)**, factor IX **(B)**, protein C **(C)**, and angle **(D)** were measured by chromogenic assays, ELISA or TEG machines. The data were presented as mean ± SEM. The numbers of analyzed patients for the individual groups are displayed below the graph on each panel. Statistical analyses were performed by linear mixed-effect model for repeated measures and the least square means of each group comparison. Statistical analyses were performed using a linear mixed-effect model for repeated measures and the least square means of each group comparison. * p<0.05, ISS>24 vs. ISS<16; † *p*<0.05, ISS>25 vs. ISS=16-24. # p<0.05, ISS=16-24 vs. ISS<16.

Supplementary Figure 2 Receiver operator characteristic (ROC) curves of complement, endothelial, and coagulation markers predict clinical outcomes. ROC curves were plotted for evaluating the complement factors of Bb, C5b-9, and C3a **(A)**, endothelial markers of syndecan-1 and sTM **(B)**, and coagulation parameters of D-dimer, PTT, PT, and LY30 **(C)** in clinical outcomes of MOF/death. The areas under the ROC curves (AUC) were presented in each panel.

Supplementary Figure 3 Receiver operator characteristic (ROC) for predicting the cut-off value of Bb, syndecan-1, and D-dimer associated with muti-organ failure (MOF)/death. ROC curves were plotted for evaluating the complement factors of Bb **(A)**, endothelial markers of Syndecan-1 **(B)**, and coagulation parameter of D-dimer **(C)** in clinical outcomes of MOF/death. The areas under the ROC curves (AUC) were presented in each panel.
