# Supplementary figures and images for: Traumatized triad of complementopathy, endotheliopathy, and coagulopathy ˗ Impact on clinical outcomes in severe polytrauma patients
Source: Front Immunol. 2022 Oct 20;13:991048. doi: 10.3389/fimmu.2022.991048 (PMC9632416; doi:10.3389/fimmu.2022.991048)

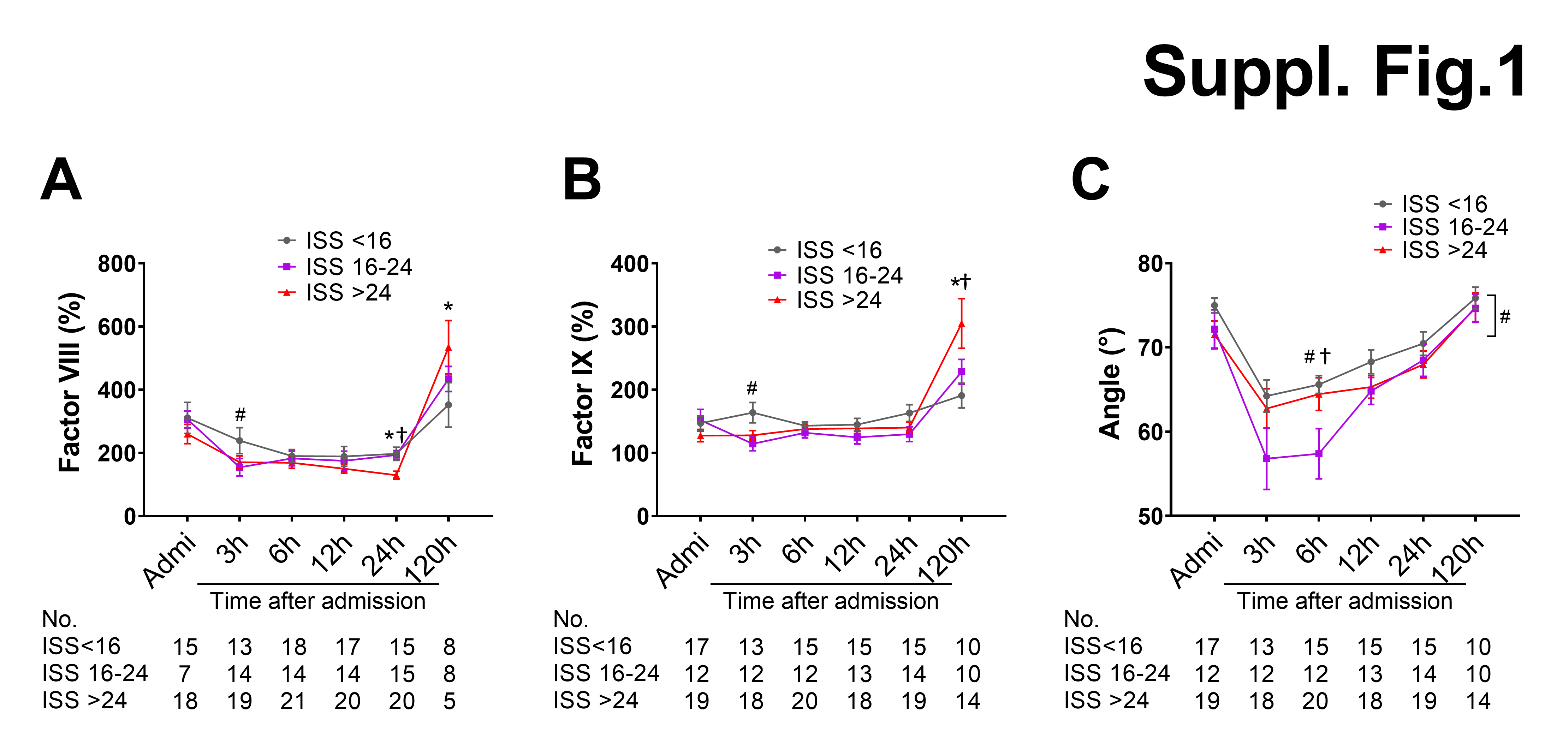

Supplement: Supplementary Figure 1 — Coagulation and fibrinolysis parameters abnormalities in poly-trauma patients. The blood samples were collected upon admission and at 3h, 6h, 12h, 24h, and 120h after admission, and the coagulation and fibrinolysis parameters of factor VIII (A), factor IX (B), protein C (C), and angle (D) were measured by chromogenic assays, ELISA or TEG machines. The data were presented as mean ± SEM. The numbers of analyzed patients for the individual groups are displayed below the graph on each panel. Statistical analyses were performed by linear mixed-effect model for repeated measures and the least square means of each group comparison. Statistical analyses were performed using a linear mixed-effect model for repeated measures and the least square means of each group comparison. * p<0.05, ISS>24 vs. ISS<16; † p<0.05, ISS>25 vs. ISS=16-24. # p<0.05, ISS=16-24 vs. ISS<16. [file Image_1.tif]

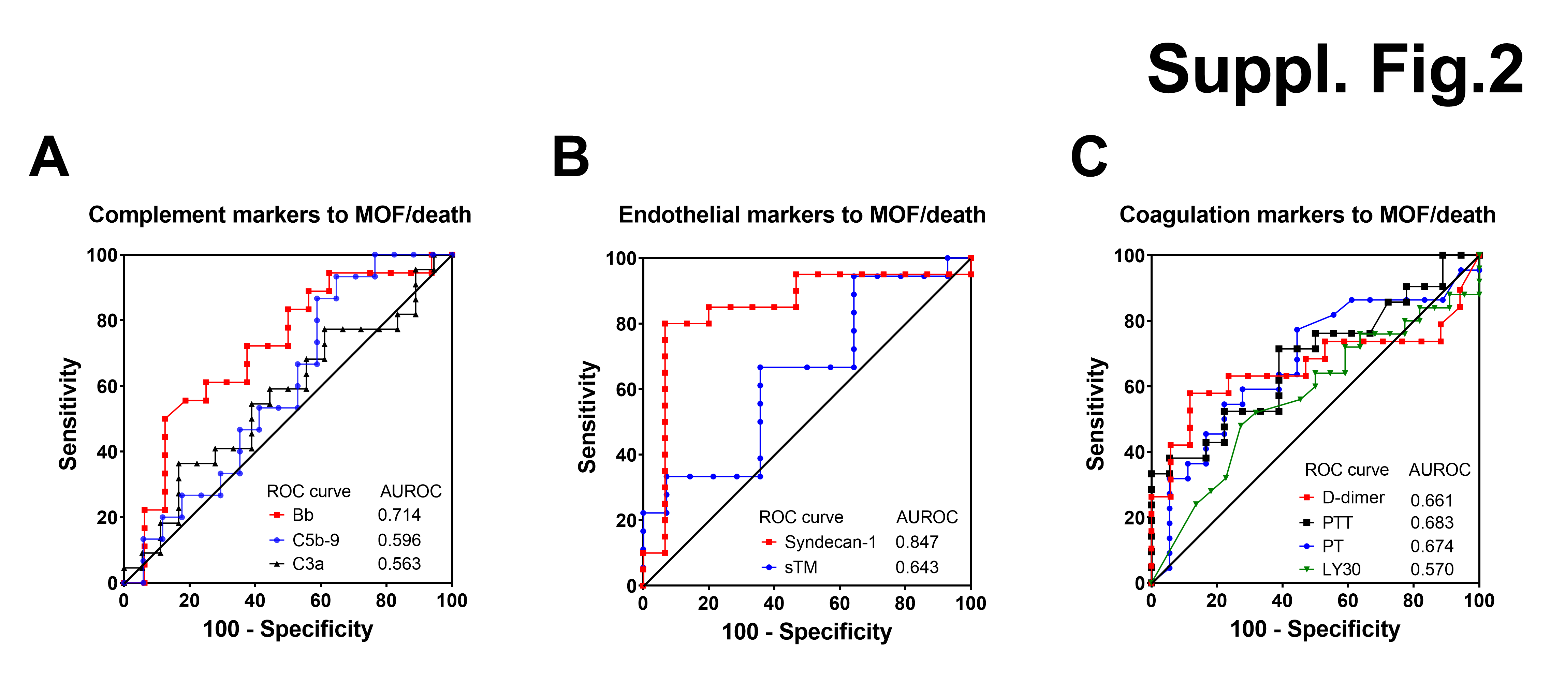

Supplement: Supplementary Figure 2 — Receiver operator characteristic (ROC) curves of complement, endothelial, and coagulation markers predict clinical outcomes. ROC curves were plotted for evaluating the complement factors of Bb, C5b-9, and C3a (A), endothelial markers of syndecan-1 and sTM (B), and coagulation parameters of D-dimer, PTT, PT, and LY30 (C) in clinical outcomes of MOF/death. The areas under the ROC curves (AUC) were presented in each panel. [file Image_2.tif]

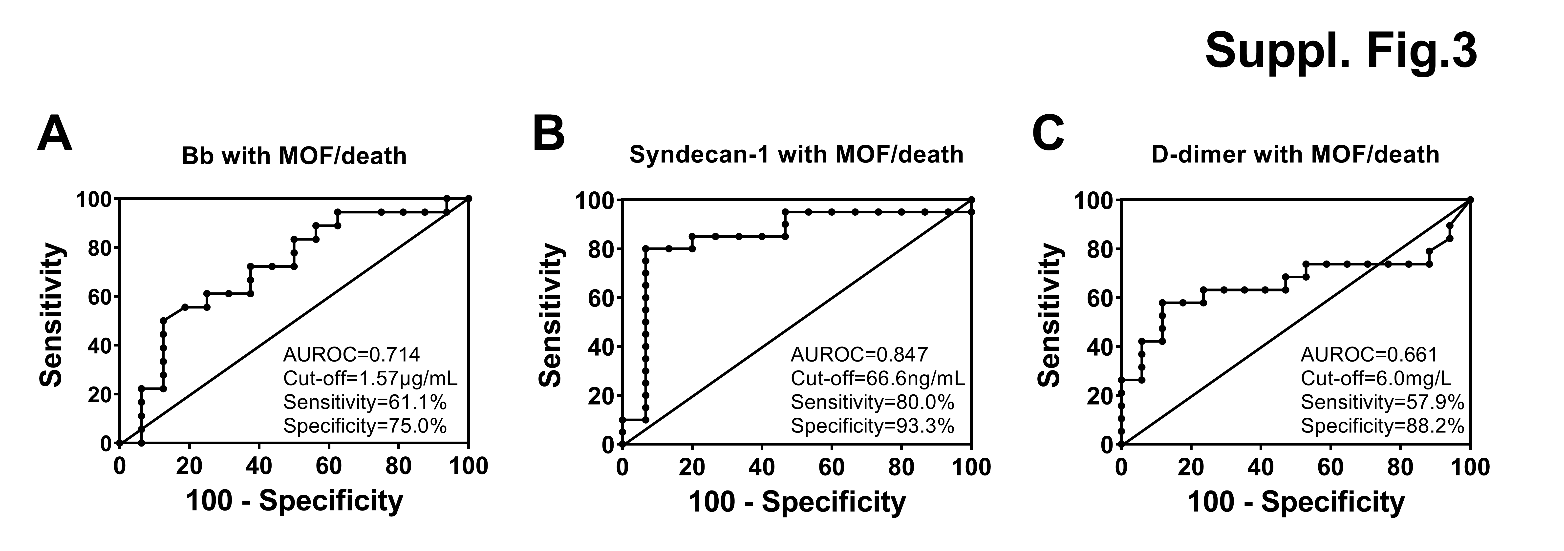

Supplement: Supplementary Figure 3 — Receiver operator characteristic (ROC) for predicting the cut-off value of Bb, syndecan-1, and D-dimer associated with muti-organ failure (MOF)/death. ROC curves were plotted for evaluating the complement factors of Bb (A), endothelial markers of Syndecan-1 (B), and coagulation parameter of D-dimer (C) in clinical outcomes of MOF/death. The areas under the ROC curves (AUC) were presented in each panel. [file Image_3.tif]
